# Making Sense of the Growth Behavior of Ultra-High Magnetic Gd_2_-Doped Silicon Clusters

**DOI:** 10.3390/molecules28135071

**Published:** 2023-06-28

**Authors:** Biao Xie, Huai-Qian Wang, Hui-Fang Li, Jia-Ming Zhang, Jin-Kun Zeng, Xun-Jie Mei, Yong-Hang Zhang, Hao Zheng, Lan-Xin Qin

**Affiliations:** 1 College of Information Science and Engineering, Huaqiao University, Xiamen 361021, China; 2 College of Engineering, Huaqiao University, Quanzhou 362021, China

**Keywords:** density functional theory, electronic property, photoelectron spectroscopy, cluster, structural evolution

## Abstract

The growth behavior, stability, electronic and magnetic properties of the Gd_2_Si_n_^−^ (n = 3–12) clusters are reported, which are investigated using density functional theory calculations combined with the Saunders ‘Kick’ and the Artificial Bee Colony algorithm. The lowest-lying structures of Gd_2_Si_n_^−^ (n = 3–12) are all exohedral structures with two Gd atoms face-capping the Si_n_ frameworks. Results show that the pentagonal bipyramid (PB) shape is the basic framework for the nascent growth process of the present clusters, and forming the PB structure begins with n = 5. The Gd_2_Si_5_^−^ is the potential magic cluster due to significantly higher average binding energies and second order difference energies, which can also be further verified by localized orbital locator and adaptive natural density partitioning methods. Moreover, the localized f-electron can be observed by natural atomic orbital analysis, implying that these electrons are not affected by the pure silicon atoms and scarcely participate in bonding. Hence, the implantation of these elements into a silicon substrate could present a potential alternative strategy for designing and synthesizing rare earth magnetic silicon-based materials.

## 1. Introduction

The developments of nanoclusters not only exhibit size-independent properties but also provide a rational tool to “design” the tailored physical and chemical properties. Hence, the research on this aspect has achieved great success in the past few decades [1,2,3,4,5,6]. As the foundation of modern industry, Si plays an important role in electronic materials, nanomaterials and self-assembled materials. In particular, small silicon clusters are promising for the blocks of nanometric materials; consequently, silicon-based clusters have drawn wide attention and have been investigated extensively between experiment [7,8,9,10] and theory [11,12,13,14]. Unfortunately, the presence of dangling bonds on the clusters makes the pure silicon clusters unstable [15,16], which is unsuitable for building blocks. Unlike carbon atoms, silicon atoms prefer the sp3 hybridization to sp^2^ [17] because the π bonding in silicon is much weaker than in carbon. Although both C and Si have diamond crystal structures, there is no silicon solid in the graphite phase, and the hollow cage structures in silicon clusters are usually unstable. Therefore, silicon-based clusters are both unstable and unsuitable as a unit for modern nanomaterials.

Since the discovery that the “impurity” could shed light on silicon-based clusters with particular properties and enhanced stability, especially for metal-doped silicon clusters, many investigations on doped silicon clusters have been reported [18,19,20,21,22,23,24]. In pioneering studies in 1987 [25] and 1989 [26], Beck carried out experiments on metal-doped silicon clusters, especially with the addition of transition metal™, and found that the structure of closed shell usually brings higher stability. Kumar et al. [11] reported the three new classes of metal-encapsulated M@Si_n_ clusters with high stability and large HOMO-LUMO gaps; a large gap was observed for Ti@Si_16_ Frank-Kasper polyhedron. Combined mass spectra with photoelectron spectra, Koyasu et al. [10] determined the formation of metal atom encapsulated in a Si cage MSi_16_ (M = Sc, Ti and V); it was found that the magnitude HOMO-LUMO gap for TiSi_16_ is the largest among those measured for single metal-atom-doped silicon clusters. A Frank–Kasper polyhedron was also predicted by their calculations. However, magnetic moments tend to quench in metal-atom-doped silicon-based clusters owing to the strong hybridization between metal-3d and Si-3p orbital [27,28,29,30].

In contrast to 3d TM atoms, 4f-orbital electrons are well preserved and localized at both lanthanide atoms. Thus, intensive research on lanthanide-atom-doped silicon clusters has been carried out in recent years on silicon clusters [31,32,33]. Grubisic et al. [34] published the photoelectron spectra of EuSi_n_ cluster anions, and EuSi_12_ was measured to be the smallest fully endohedral europium-silicon cluster. Nguyen and co-workers investigated the magnetic properties and geometric and electronic structures of Si_7_M^0/−1^ with M = Pr, Gd, Ho [35], and the limited f-electrons of the lanthanide metal atoms were observed by density functional theory (DFT). As a consequence, high magnetic moments were induced in doped clusters. Motivated by these, we set out to systematically probe the structural, spectral, electronic and bonding properties of Gd_2_Si_n_^−^ (n = 3–12), expanding the investigation of Gd-diatom doped silicon systems. The aim of this work is (1) to obtain the various global minimum structures of Gd_2_Si_n_^−^ (n = 3–12); (2) to pursue the growth behavior of Gd_2_Si_n_^−^ (n = 3–12); (3) to understand the effect of the doping of two Gd atoms on the electronic properties; and (4) to explore the interaction mechanism between Gd and pure silicon atoms. We hope that the present work will greatly deepen understanding of these diatomic lanthanide systems, and as promising building blocks in developing cluster-assembled materials.

## 2. Results and Discussion 

**Figure 1:**
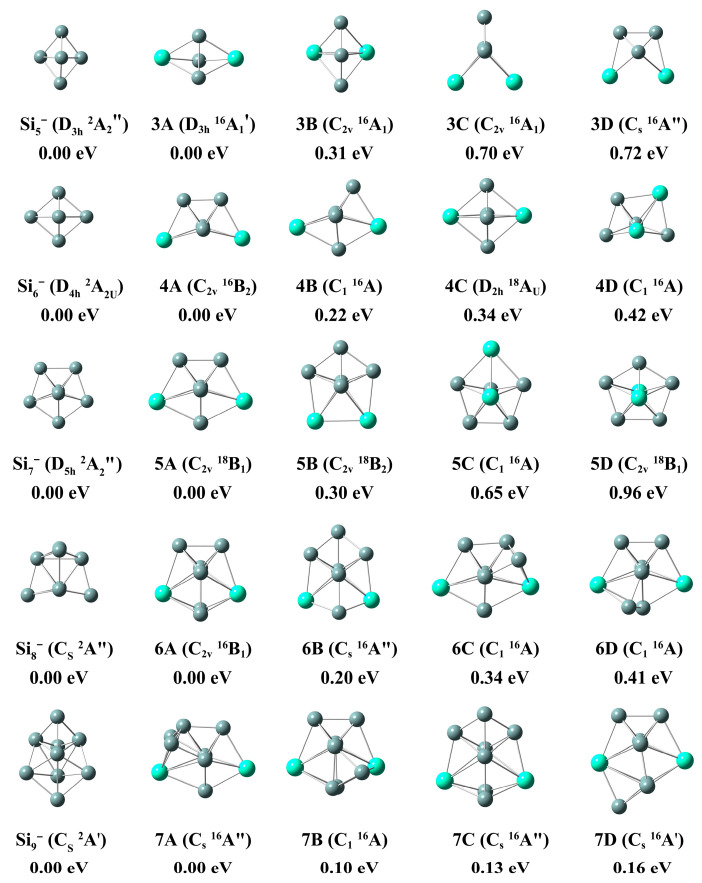
The global minimum and low-energy isomers of Gd_2_Si_n_^−^ and Si_n+2_^−^ (n = 3–7) with relative energy, symmetry and electron state at the PBE0/Gd/ECP28MWB//Si/6-311+G(d) level. The gray and light green balls represent silicon and gadolinium atoms, respectively.

**Figure 2:**
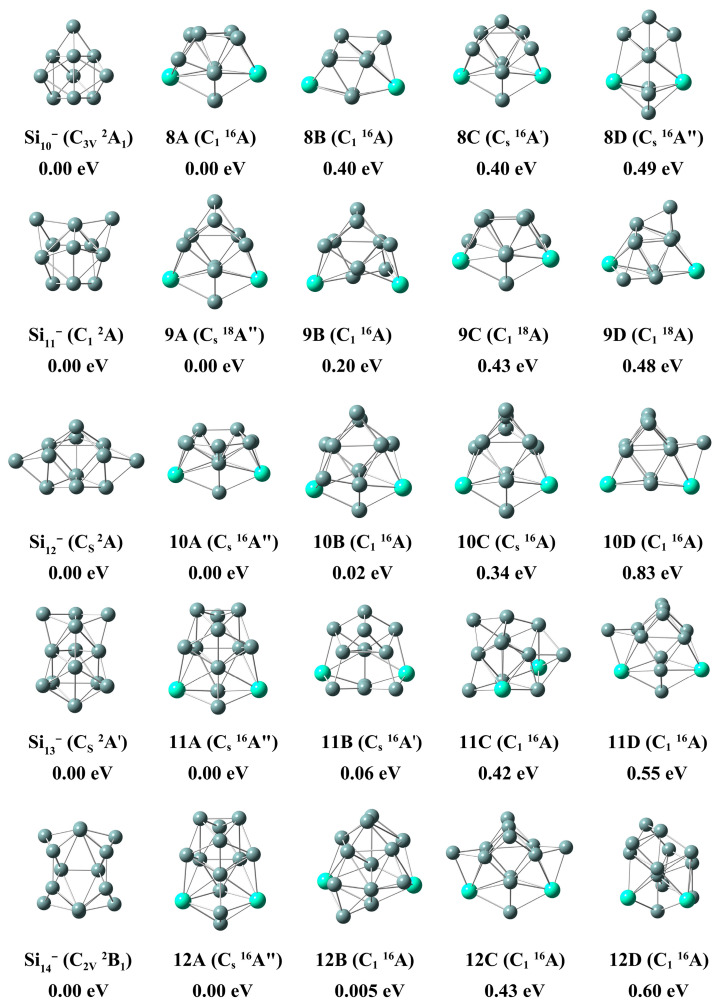
The global minimum and low-energy isomers of Gd_2_Si_n_^−^ and Si_n+2_^−^ (n = 8–12) with relative energy, symmetry and electron state at the PBE0/Gd/ECP28MWB//Si/6-311+G(d) level. The gray and light green balls represent silicon and gadolinium atoms, respectively.

The top several optimized lowest-lying isomers of Si_n+2_^−^ and Gd_2_Si_n_^−^ (n = 3–12) clusters are depicted in Figure 1 and Figure 2, and more information on isomeric structures is depicted in Figures S2 and S3 (see Supplementary Materials). The Cartesian coordinates of the top four isomeric structures are summarized in Table S2 of the Supplementary Material. The detailed descriptions of the geometric structures are shown below.

### 2.1. Geometric Structures

In this section, we discuss the structural details of some low-lying isomers of Gd_2_Si_n_^−^ clusters. Here, in order to discuss the effects of doped bimetallic Gd atoms on pure silicon clusters, the structures of silicon clusters are also plotted in Figure 1 and Figure 2 for comparison, and are consistent with previous work [36,37,38]. The detailed spin multiplicity (SM), the symmetry (Sym), the relative energy (∆E), the first adiabatic detachment energy (ADE) and the first vertical detachment energy (VDE) for the low-lying isomers of Gd_2_Si_n_^−^ (n = 3–12) clusters are shown in Table S3.

#### 2.1.1. Si_5-8_^−^ and Gd_2_Si_3-6_^−^

Compared to trigonal bipyramid-shaped Si_5_^−^, as for Gd_2_Si_3_^−^, the lowest-lying isomer 3A (D_3h_, ^16^A_1_′) can be obtained by the two Gd atoms substituting two Si atoms located at the vertex of top and bottom sides. The first ADE and VDE for 3A are 1.20 and 1.37 eV, respectively. Si_6_^−^ is predicted to be a square bipyramid-shaped structure with D_4h_ symmetry. Isomer 4A can be regarded as adding a Si atom to the vertex of 3A, forming a distorted tetragonal bipyramid pattern structure with C_1_ point group symmetry and ^16^A electron state. The computed first ADE and VDE are 1.01 and 1.09 eV, respectively. The pentagonal bipyramid-shaped structure of Si_7_^−^ with a more symmetric configuration (D_5h_) is presented in Figure 1. Meanwhile, analogous to pure Si_7_^−^, the most stable isomer of Gd_2_Si_5_^−^ was also found to be a pentagonal bipyramid (PB) with C_2v_ symmetry and ^18^B_1_ electron state. Substituting two Si atoms on the D_5h_ planar of the decahedron with two Gd atoms, the first ADE and VDE values of 5A (0.78 and 0.84 eV) were obtained at the same level of calculation. In contrast to pure crown-shaped Si_8_^−^ (C_s_, ^2^A″), the most stable Gd_2_-doped 6A can be obtained by adding a Si atom to face-cap the bottom of 5A coupled with C_2v_ symmetry and ^16^B_1_ electron state; the first ADE and VDE are 1.95 and 2.09 eV, respectively.

#### 2.1.2. Si_9-14_^−^ and Gd_2_Si_7-12_^−^

With respect to Gd_2_Si_7_^−^, the top-four isomers (7A, 7B, 7C and 7D, relative energy within ~0.20 eV) all emerged as the candidates, as shown in Figure 1 at PBE0/Gd/ECP28 MWB//Si/6-311+G(d) level. The ground state structure of isomer 7A can be described as putting two Si atoms on the top left side of PB (5A) with C_s_ point group symmetry and 16A″, and it also can be seen as attaching a Si atom to the top left of 6C. Both the computed ADE and VDE (2.16 eV for ADE and 2.20 eV for VDE) of 7A are measured in this work, suggesting that the anionic structure is essentially unchanged by the loss of an electron. Si_10_^−^ is of C_3v_ symmetry with a fruit basket-shaped structure. It was found that 8A is based on the 5A and three Si which connect to PB, with C_1_ symmetry and ^16^A electron state, and the ADE and VDE values of 8A are 1.77 and 2.19 eV, respectively. Comparing the low symmetric structure of Si_11_^−^, the lowest-lying stable geometrical structure of 9A is of C_s_ symmetry with ^18^A″ electron state, which can be generated based on 8C. Appending an excess Si atom to the apex of 8C, eleven atoms were separated into two parts (one of which was a PB and the other was a triangular pyramid). The computed ADE and VDE values of 9A are 1.75 and 1.86 eV. Si_12_^−^ is predicted to be a teapot-shaped geometry with C_s_ symmetry. With respect to Gd_2_Si_10_^−^ cluster, isomer 10A was generated when capping the five-atom pentagon on the top of 5A, with C_s_ point group symmetry and ^16^A electron state. The theoretical ADE and VDE values of 10A are 2.26 and 2.31 eV. As far as the Gd_2_Si_11_^−^ clusters are concerned, the lowest-lying stable geometry was generated when two additional Si capped the top side of 9A, which can also be depicted as the combination between a Si_6_ with a pentagonal bipyramid-shaped Gd_2_Si_5_. Its computed ADE and VDE values are 2.31 and 2.54 eV, respectively. Compared to pure Si_14_^−^ (C_2V_, ^2^A) cluster, the lowest-lying structure 12A can be considered as appending an additional Si atom at the bottom of 11A, and also can be seen capping seven Si atoms on the framework of PB. The values of ADE and VDE for ground state Gd_2_Si_12_^−^ are 2.53 and 2.85 eV.

### 2.2. The Growth Behavior of Gd_2_Si_n_^−^

It is interesting to probe the structural evolution, and it can be found that the lowest-lying structures of Gd_2_Si_n_^−^, with n = 5–12, are all exohedral structures. The optimized structures show that the ground states of clusters favor high spin state. It is strongly worth mentioning that the PB is the basic framework for the growing size of Gd_2_Si_n_^−^ (n = 3–12), as presented in Figure S4. Gd_2_Si_n_^−^ still can be regarded as replacing two Si atoms with two Gd atoms, the basic framework of PB initially formed in n = 5. For n = 6–12, all clusters can be viewed as absorbing one to seven Si atoms on the different positions of PB; each of the ground-state structures retained the motif and grew on it. However, they cannot simply be seen as occupying the original silicon-based clusters. Compared to previous investigations of T_2_Si_n_^−1/0^ (T = Fe, Co and Ni, 1 ≤ n ≤ 8) [39], T_2_Si_n_ (T = Cr, Mn, 1 ≤ n ≤ 8) [40], Au_2_Si_n_^−1/0^ (n = 1–7) [41], Nb_2_Si_n_ (n = 2–12) [42], Mo_2_Si_n_ (n = 9–16) [43] and Fe_2_Si_n_ (n = 1–12)^+/0/−^ [44], the two TMs tend to form a strong metal–metal bond, which is different from the weak interaction between Gd-Gd, implying the bonding properties of two Gd-atom-doped silicon clusters are different from those silicon clusters doped with other TMs. On the one hand, the early formation of half-endohedral geometric structures appears at n ≥ 9; one of the metal atoms is embedded in the cage while the other metal atom tends to adsorb on the surface. It is interesting to compare the growth behaviors with the corresponding bimetal-doped Si clusters counterparts; both of the Gd atoms always adsorb on the surface of Si-based clusters. The reason for this is probably because Gd atoms have a larger atomic radius and are more difficult to embed into the silicon cage. On the other hand, the infrequent interaction between dual Gd reveals that they are inclined to adsorb the surface instead of being embedded in a Si cage.

### 2.3. Simulated Photoelectron Spectrum 

**Figure 3:**
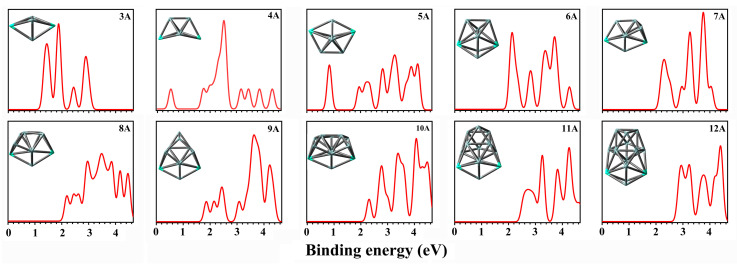
The simulated photoelectron spectra of lowest energy isomers of Gd_2_Si_n_^−^ (n = 3–12) at the PBE0/Gd/ECP28MWB//Si/6-311+G(d) level. The simulated photoelectron spectra exhibit a full width at half maximum (FWHM) of 0.20 eV.

In order to provide theoretical guidance for further experiments, we have generated simulated photoelectron spectra (PESs) for the lowest-lying isomers of Gd_2_Si_n_^−^ (n = 3–12), as illustrated in Figure 3. The simulated PESs were fitted by adding the energy of the occupied orbital to the VDE, based on the generalized Koopman’s theorem (GKT) [45], with a full width at half maximum (FWHM) value of 0.20 eV. Additionally, more simulated PESs of low-lying isomers can be obtained in Figure S5.

In Figure 3, the PESs of Gd_2_Si_n_^−^ (n = 3–12) reveal unique features. The first peak of the spectrum of Gd_2_Si_3_^−^ is denoted as the first VDE, centered at 1.44 eV, and followed by three distinct features at 1.87, 2.44 and 2.89 eV, respectively. Similarly, Gd_2_Si_4_^−^ shows a small peak at 1.91 eV, followed by four spectrum features located at 1.36, 2.30, 2.96 and 3.49 eV, respectively. As for the spectrum of Gd_2_Si_5_^−^, it shows a very broad feature extending from approximated 0.8–4.2 eV, furthermore, six sharp peaks can be observed at 0.84, 1.95, 2.83, 3.27, 3.89 and 4.13 eV, respectively. There are five obvious peaks centered at 2.14, 2.83, 3.37, 3.72 and 4.27 eV that can be detected in the spectrum of Gd_2_Si_6_^−^. The spectrum of Gd_2_Si_7_^−^ displays three prominent peaks located at 2.30, 3.26 and 3.75 eV, along with two small peaks centered at 2.96 and 4.04 eV. The spectrum of Gd_2_Si_8_^−^ shows six sharp peaks centered at 2.19, 2.96, 3.48, 3.85 and 4.17 eV, followed by two small peaks located at 2.45 and 2.62 eV, respectively. There are six distinguishable features at 1.86, 2.15, 2.44, 3.08, 3.64 and 4.21 eV that can be detected in the spectrum of Gd_2_Si_9_^−^. The spectrum of Gd_2_Si_10_^−^, displays five obvious features at 2.31, 2.78, 3.39, 4.07 and 4.48 eV. Three peaks can be observed at higher electron binding energy regions centered at 3.26, 3.82 and 4.25 eV, which are distinguishable in the spectrum of Gd_2_Si_11_^−^. Finally, the spectrum of Gd_2_Si_12_^−^ shows four distinct peaks located at 2.94, 3.24, 3.77 and 4.39 eV.

### 2.4. Relative Stability

To measure the relative stability of bimetallic Gd doped silicon clusters, the average binding energy and second order difference energy of the lowest energy structures for Gd_2_Si_n_^−^ are calculated at PBE0/Si/6-311+G(d)//Gd/ECP28MWB level for Gd_2_Si_n_^−^ and the PBE0/Si/6-311+G(d) level for Si_n+2_. The results of average binding energy are shown in Figure S6.

The average binding energy and second order difference energy are defined by the following formulas:

E_b_ (Gd_2_Si_n_^−^) = [(n − 1) E(Si) + E(Si^−^) + 2 E(Gd) − E(Gd_2_Si_n_^−^)]/n + 2 (1)

E_b_ (Si_n+2_^−^) = [(n + 1) E(Si) + E(Si^−^) − E(Si_n+2_^−^)]/n + 2 (2)

∆^2^E(Gd_2_Si_n_^−^) = E(Gd_2_Si_n+1_^−^) + E(Gd_2_Si_n-1_^−^) − 2E(Gd_2_Si_n_^−^) (3)

∆^2^E(Si_n+2_^−^) = E(Si_n+3_^−^) + E(Si_n+1_^−^) − 2E(Si_n+2_^−^) (4)

where the E(Gd_2_Si_n+1_^−^), E(Gd_2_Si_n_^−^), E(Gd_2_Si_n-1_^−^), E(Si_n+3_^−^), E(Si_n+2_^−^), E(Si_n+1_^−^) represent the total energy of Gd_2_Si_n+1_^−^, Gd_2_Si_n_^−^, Gd_2_Si_n-1_^−^, Si_n+3_^−^, Si_n+2_^−^, Si_n+1_^−^ in ground state, respectively. E(Si^−^), E(Si) and E(Gd) correspond to the single-point energies of Gd and Si atoms.

#### 2.4.1. Average Binding Energy

Figure S6 shows the average binding energy of ground-state structures of Gd_2_Si_n_^−^ (n = 3–12) at the PBE0/Gd/ECP28MWB//Si/6-311+G(d) level. There are several observations worth noting from Figure S6.

(1) The binding energy of pure Si_n+2_^−^ and mixed Gd_2_Si_n_^−^ clusters both rise monotonically with increasing dimensions of growing size; however, the binding energy per atom for Si_n+2_^−^ is slightly higher than Gd_2_Si_n_^−^, suggesting that the stability of two Gd atoms doping is smaller than Si_n+2_^−^.
(2) It is interesting to find that Gd_2_Si_5_^−^ and Gd_2_Si_8_^−^ have a relatively steep upward trend along with increasing dimensions of growing size, signifying that Gd_2_Si_5_^−^ and Gd_2_Si_8_^−^ are the most stable clusters in the range researched here.

#### 2.4.2. Second Order Difference Energy

**Figure 4:**
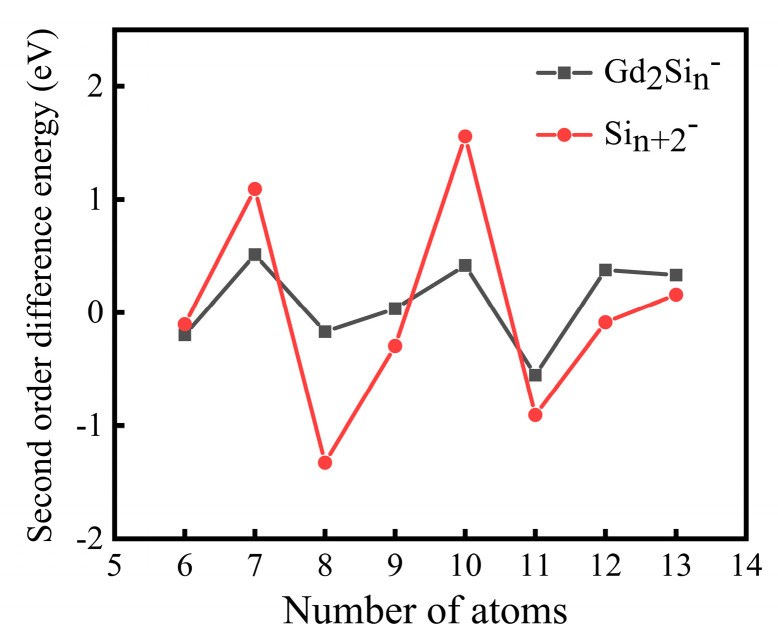
The second order difference energies of Gd_2_Si_n_^−^ (n = 4–11) and Si_n+2_^−^ at PBE0 /Gd/ECP28MWB//Si/6-311+G(d) level.

The second order difference energy ∆^2^E for Si_n+2_^−^ and Gd_2_Si_n_^−^ is presented in Figure 4. As a sensitive indicator to measure the relative stability of clusters in comparison with their smaller and larger neighbors, ∆^2^E gives the evidence to identify the most stable clusters by the maximum values. The primary features are concluded below:

(1) The second difference energy of Si_n+2_^−^ and Gd_2_Si_n_^−^ as a function of the cluster size exhibits a pronounced even–odd alternation phenomenon.
(2) The relative stabilities of Gd_2_Si_5_^−^ and Gd_2_Si_8_^−^ are quite strong among all the clusters in terms of second order difference energy.
(3) We could easily screen out the magic clusters (Gd_2_Si_5_^−^) with the help of results of second order difference energy.

### 2.5. Magnetic Properties and Natural Atomic Orbital

In order to comprehend the interaction between Gd atoms and silicon-based clusters, we performed natural atomic orbital (NAO) for the lowest-energy structures of Gd_2_Si_n_^−^ cluster using PBE0 functional. The natural population analysis (NPA) charges of the lowest-energy structures of Gd_2_Si_n_^−^ (n = 3–12) are given in Table S4. As shown in Table S4, charges always transfer from the two Gd atoms to Si atoms in the system of increasing size. This indicates that the two Gd atoms act as donors while the Si atoms act as receptors, reflecting the bonding process. This is further supported by the valence electron configurations and magnetic moments given in Table S5. It is well known that the valence electron configuration of a free Gd atom is [Xe]4f^7^5d^1^6s^2^. Based on the data in Table S5, two conclusions can be drawn. Firstly, both Gd atoms still possess half-filled 4f electrons, which is quite different from dual TMs doping [39,40,44], suggesting the hard participation in bonding between Si atoms; therefore, it has a very good prospect in the application of Si-based cluster-assembled materials. Secondly, the transferred 6s and 5d electrons reveal that the interaction among 6s and 5d orbitals can be proposed. This conclusion is discussed in detail below. 

Gd-4f electrons to a large extent appear not to interact significantly with their neighboring Si atoms, as demonstrated by the distribution of single spin electron population of Gd_2_Si_n_^−^ (n = 3–12) in Figure S7. Obviously, the excess electrons are mainly distributed around the Gd atoms, as indicated by the red region, which confirm that the high magnetic moments are derived from the two Gd atoms, visually. Additionally, the red dense isosurface can be inferred mainly to distribute a bunch of 4f electrons with the help of NAO analysis, and then the results of spin density isosurface more strongly confirm the distribution of bimetallic Gd among the mixed system.

### 2.6. Bonding Analysis

**Figure 5:**
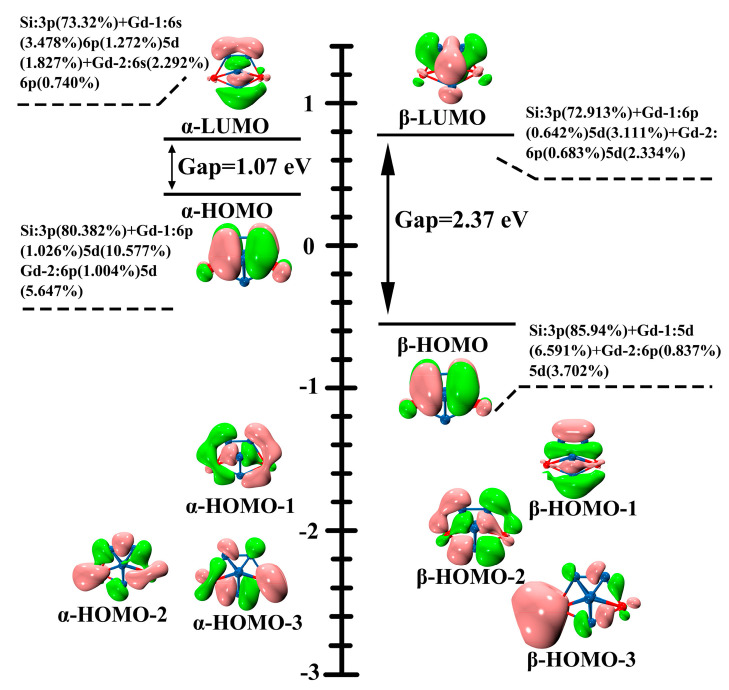
Valence MOs and the corresponding energy levels of Gd_2_Si_5_^−^, in conjunction with molecular orbital composition and SOMO-LUMO gap is indicated (in black).

In order to shed some light on the origins of the high stability of Gd_2_Si_5_^−^, the compositions of their molecular orbitals (MOs) in the vicinity of the frontier MOs of the Gd_2_Si_5_^−^ are plotted in Figure 5 to research stability mechanisms. The frontier orbital energy gaps can be regarded as a distinguishing feature of kinetic stability of chemical compounds. In addition, high reluctance of compounds for chemical reactions also can be elaborated by a large gap. It can be seen that the identical frontier molecular orbitals (α-HOMO, α-LUMO, β-HOMO, and β-LUMO) are mainly derived from Si-3p and Gd-6s 6p 5d. In particular, the 5d orbital of Gd atom can be seen as the main participant in α-HOMO, α-LUMO, β-HOMO, and β-LUMO orbitals; that is, the distinctive p-d hybridization makes Gd_2_Si_5_^−^ possess high stability. Furthermore, the higher HOMO-LUMO gap also can be observed in Figure 5. The large gaps at both alpha (1.07 eV) and beta (2.37 eV) orbitals can be expected to give rise to the stability of Gd_2_Si_5_^−^, which gives further support for this enhanced stability.

**Figure 6:**
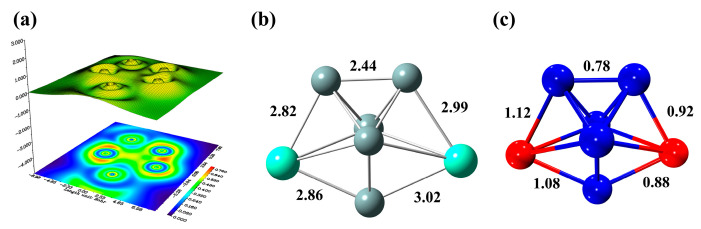
The color-filled map of LOL (**a**), bond length (**b**) and fuzzy bond order (**c**) of global minimum structure of Gd_2_Si_5_^−^ at PBE0/Gd/ECP28MWB//Si/6-311+G(d) level.

With the purpose to characterize the Si-Gd and Si-Si bond, the localized orbital locator (LOL) is adopted for double Gd-atom-doped silicon clusters, which is plotted in Figure 6a. As a tool for classification of chemical bonding, atomic shell structure and electronic structure, LOL has been widely applied for organic or inorganic small molecules, atomic crystals, clusters and coordination compounds, which helps us better understand the strength of the interaction between atoms. It is significant to gain deep insight into the bonding property of Gd_2_Si_5_^−^. The mean electronic population is distinguished by different colors. The regions with high LOL values indicate more localized electrons and are of great interest in chemical bonding. As can be seen from Figure 6a, the high LOL values are determined between Si_1_, Si_4_ and Si_3_, suggesting that the Si-Si covalent interaction can be intuitively identified based on the red region and the electrons are remarkably limited between the Si atoms. Furthermore, it is worth mentioning that the distinct red region can be observed at Figure 6a, indicating that the localized Si-Gd bonding is accountable for the interaction between double Gd dopant and Si moiety. This can be validated by bond length and fuzzy bond order, as shown in Figure 6b,c, and it is found that both high values of fuzzy bond for Si-Si and Si-Gd can be intuitively visualized and indeed this case can be supported by bond length. To summarize, the high stability of Gd_2_Si_5_^−^ is attributed to the interaction between Si atoms in the host silicon cluster. Furthermore, the enhanced stability of the Gd-doped silicon cluster is derived from the strong interaction between Si atoms and the Gd dopant.

**Figure 7:**
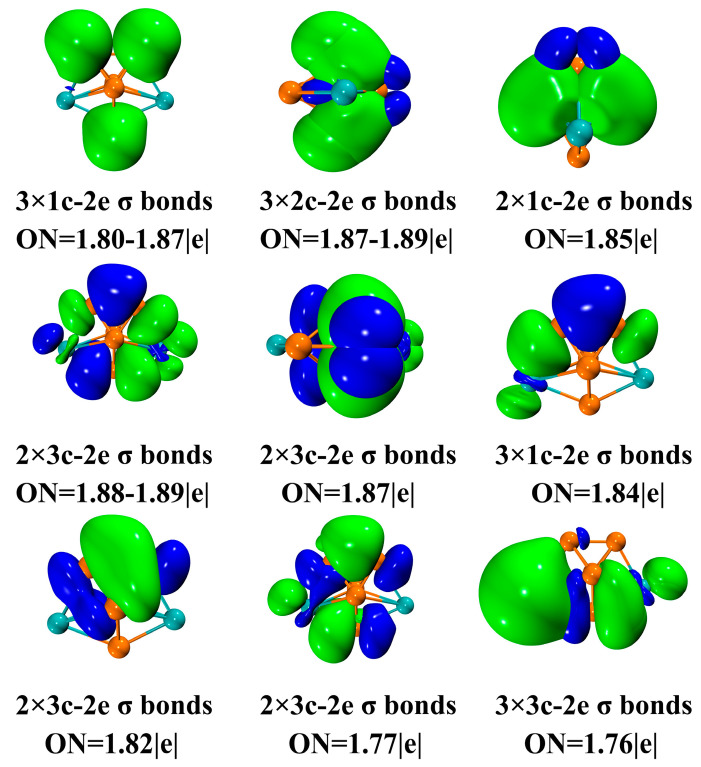
Analysis of the AdNDP chemical bonds for Gd_2_Si_5_^−^.

The analysis of AdNDP results also gives insight into the multicenter bond (nc-2e) and delocalization of Gd_2_Si_5_^−^ (as shown in Figure 7). Moreover, the AdNDP results provide compelling evidence to support the conclusion regarding the stability of Gd_2_Si_5_^−^. The effect of mixing two Gd atoms on the chemical bonding could be further elaborated in current work. AdNDP is based on natural atomic orbital (NAO), which is defined as the diagonalization of the density matrix of the *n*-atomic sub-block of *N*-atomic molecular system. The three (1c-2e) lone pairs are responsible for the impact on the geometric structure, where localized electrons are concentrated on three Si atoms that surround the plane of the pentagonal bipyramid of the ground-state structure for Gd_2_Si_5_^−^. It should be noted that five 2c-2e Si-Si σ bonds could be observed in Figure 7 while these 2c-2e σ bonds all concentrated on Si atoms with ON = 1.85–1.89|e|, indicating the strong covalent bonding could be observed among Si atoms. This observation is consistent with the LOL analysis, bond length and fuzzy bond order analysis (see Figure 6), mentioned above, which provides a good illustration of the existence of covalent bonds with high LOL values. Furthermore, the shorter bond length on Gd-Si incorporated with a high value of fuzzy bond order also confirms the strong Gd-Si interaction. The remaining delocalized π bonds can be characterized on twelve 3c-2e AdNDP orbitals (1.76–1.89|e|). Most interestingly, both two Gd atoms can almost be seen in delocalized bonds, which explains the strong stability of Gd_2_Si_5_^−^.

## 3. Computational Methods

All the calculations of equilibrium geometries of Gd_2_Si_n_^−^ (n = 3–12) are carried out by DFT with PBE0 functional [46], as implemented in GAUSSIAN 09 program package [47]. They are all optimized until the harmonic vibration analysis gives no imaginary frequency. The introduction of the two Gd atoms makes the search even more difficult; for small clusters, this problem can be solved by using the Saunders “Kick” (SK) global search technique [48]. However, as the size increases, the number of local minimal isomers increases exponentially; how to search the initial structures quickly and comprehensively is a difficult problem. In addition to the SK stochastic method, we also employed the Artificial Bee Colony cluster (ABC) [49] algorithm to search global minimum and low-lying structures for larger clusters. Two global optimization schemes (SK and ABC) complement each other to obtain the real global optimal structure. Our group has successfully performed the investigations of binary clusters by these methods [50,51,52,53,54,55].

During the pre-optimization, about 300–600 isomeric structures for Gd_2_Si_n_^−^ (number of atoms less than 8) and 600–1000 structures for Gd_2_Si_n_^−^ (number of atoms more than 8) were generated with unbiased search and optimized at PBE0 functional with the 6-31G basis set for Si atoms. Due to significant relativistic effects on lanthanide elements, the larger scalar Stuttgart relativistic effective core potential basis set (ECP54MWB) [56] was chosen for Gd atoms. Furthermore, we carried out substitution and adsorption of the two Gd atoms on the basis of Si_n+2_^−^ and GdSi_n+1_^−^, respectively. Subsequently, the obtained low-lying isomers from the result of random kicking and unbiased search were reoptimized at the PBE0 level with the 6-311+G(d) [57] basis set for Si atoms and the ECP28MWB [58] basis set for Gd atoms (PBE0/Gd/ECP28MWB//Si/6-311+G(d)). Additionally, there are several points worth noting: (1) a sequence of probable spin multiplicities must be taken into consideration during the process of obtaining global minimum structures; (2) self-consistent calculations were performed with a convergence criterion of 10^−6^ Hartree on the total energy and corresponding neutral counterpart; (3) the zero-point energy (ZPE) was not considered in this work, because the ZPE correction of a specific cluster was small and almost the same and was not expected to affect the relative energy ordering. 

To test the reliability of the present computational method, we performed the calculation of GdSi_4_^−^ with five different exchange-correlation functionals (PBE0 [46], B3LYP [59], PBE [60], TPSSh [61], and BPW91 [62]) with the same basis set. The choice of functionals has a significant impact on the results of the ADE calculation and the simulation of the photoelectron spectrum. It is worth noting that the PBE0/Gd/ECP28MWB//Si/6-311+G(d) level of theory has good consistency with experimental results (Table S1 and Figure S1), and, therefore, the same level has been selected as the method of choice for Gd_2_Si*_n_*^−^. Furthermore, all kinds of wavefunction analyses, containing natural atomic orbital (NAO) [63], spin density, localized orbital locator (LOL) [64] and adaptive natural density partitioning (AdNDP) [65] theory, are conducted by multifunctional wavefunction analyzer (Multiwfn) program [66,67] and visualized by Visual Molecular Dynamics (VMD) software [68].

## 4. Conclusions

In summary, the growth behavior, stability, electronic and magnetic properties of Gd_2_Si^−^ (n = 3–12) clusters were investigated with the aid of DFT calculations. Extensive searches of the lowest-energy structures were performed by considering a number of isomeric structures. The ground-state structures of Gd_2_Si*_n_*^−^ were inclined to form three-dimensional exohedral structures. All of the results can be summarized as follows:

(1) The doped double Gd atoms do not play an important role in geometric structures in small clusters Si*_n_*^−^ (n ≤ 6), but they contribute largely to the equilibrium structures from n = 7 to n = 12. More interestingly, the PB can be observed in the basic framework for the growth process of Gd_2_Si*_n_*^−^ (n = 5–12).
(2) The result of NAO reveals that the induction of bimetallic Gd_2_ atoms provides great magnetic moments, which suggests that it may assemble magnetic semiconductor materials by using double Gd-atom-doped Si-based clusters as building blocks.
(3) According to the energetic stability, Gd_2_Si_5_^−^ is determined to the most stable cluster among the size n = 3–12. The frontier molecular orbitals also illustrate the high stability.
(4) The LOL and AdNDP reveal that the stabilization mechanism of Gd_2_Si_5_^−^ is due to strong covalent bonding interactions between Gd and Si atoms.

## Data Availability

The data that support the findings of this study are available within the article and its Supplementary Material.

## Supplementary Materials

The following supporting information can be downloaded at: https://www.mdpi.com/article/10.3390/molecules28135071/s1, Table S1: Comparison of ADEs and VDEs using different methods for GdSi_4_^−^ Table S2: Cartesian coordinates for the top low-lying isomers of Gd_2_Si_n_^−^ (n = 3–12) at the PBE0/Gd/ECP28MWB//Si/6-311+G(d) level. Table S3: Various structures with type the spin multiplicity (SM), the symmetry (Sym), the relative energy (∆E), the first adiabatic detachment energy (ADE) and first vertical detachment energy (VDE) for the low-lying isomers of Gd_2_Si_n_^−^ (n = 3–12) at the PBE0/Gd/ECP28MWB//Si/6-311+G(d) level. All energies are in eV. Table S4: The NPA charges (e) on the parent Si atoms, double-Gd atoms and total charges for the most stable isomers. Table S5: The valence electron configuration of each one of the two Gd atoms, magnetic moments (μ_B_) of the Gd-4f orbital, total magnetic moments of the two Gd atoms and total magnetic moments for the most stable isomers. Figure S1: (**a**) Photoelectron spectrum of GdSi_4_^−^ measured at 266 nm (4.66 eV), the spectrum is taken from Ref. [20]. (**b**) Simulated photoelectron spectra (PES) from the lowest-energy structures for GdSi_4_^−^ clusters at the PBE0/Gd/ECP28MWB//Si/6-311+G(d) level, together with B3LYP, PBE, TPSSh and BPW91 functionals for comparison. Each VDE was fitted with a full width at half-maximum (FWHM) of 0.20 eV to yield the simulated PES spectra. Figure S2: The various geometrical structures of Gd_2_Si_n_^−^ (n = 3–7) with relative energy, symmetry and electron state at the PBE0/Gd/ECP28MWB//Si/6-311+G(d) level. The gray and light green balls represent silicon and gadolinium atoms, respectively. Figure S3: The various geometrical structures of Gd_2_Si_n_^−^ (n = 8–12) with relative energy, symmetry and electron state at the PBE0/Gd/ECP28MWB//Si/6-311+G(d) level. The gray and light green balls represent silicon and gadolinium atoms, respectively. Figure S4. The growth behavior of Gd_2_Si_n_^−^ (n = 5–12) clusters. Figure S5: The simulated photoelectron spectra (PESs) for the assigned major clusters within 0.3 eV at the PBE0/Gd/ ECP28MWB//Si/6-311+G(d) level. The simulated PESs exhibit a full width at half maximum (FWHM) of 0.20 eV. Figure S6: The average binding energies of Gd_2_Si_n_^−^ (n = 3–12) and Si_n+2_^−^ at PBE0/Gd/ECP 28MWB//Si/6-311+G(d) level. Figure S7: The spin-density (ρ_alpha_–ρ_beta_) isosurfaces of lowest-lying isomers of Gd_2_Si_n_^−^ (n = 3–12). The isosurface is set to ±0.02. The red and blue isosurfaces show that the spin density has positive and negative values, respectively.
