# Supplementary material for: Making Sense of the Growth Behavior of Ultra-High Magnetic Gd2-Doped Silicon Clusters
Source: Molecules. 2023 Jun 28;28(13):5071. doi: 10.3390/molecules28135071 (PMC10343621; doi:10.3390/molecules28135071)
Supplement: Supplementary file 1 [file molecules-28-05071-s001.zip › molecules-2456280-supplementary.pdf]

Supplementary Materials

Making Sense of the Growth Behavior of Ultra-High Magnetic Gd_2-Doped Silicon Clusters

Biao Xie[1], Huai-Qian Wang[1, 2,*], Hui-Fang Li[2], Jia-Ming Zhang[1], Jin-Kun Zeng[1], Xun-Jie Mei[2], Yong-Hang Zhang[1], Hao Zheng[1] and Lan-Xin Qin[2]

[1] College of Information Science and Engineering, Huaqiao University, Xiamen 361021 China
[2] College of Engineering, Huaqiao University, Quanzhou 362021 China
* Correspondence: hqwang@hqu.edu.cn;

Table of Contents

Table S1. Comparison of ADEs and VDEs using different methods for $GdSi_4^-$. Page 3

Table S2. Cartesian coordinates for the top low-lying isomers of $Gd_2Si_n^-$ (n =3-12) at the PBE0/Gd/ECP28MWB//Si/6-311+G(d) level. Page 4-20

Table S3. Various structures with type the spin multiplicity (SM), the symmetry (Sym), the relative energy (ΔE), the first adiabatic detachment energy (ADE) and first vertical detachment energy (VDE) for the low-lying isomers of $Gd_2Si_n^-$ (n =3-12) at the PBE0/Gd/ ECP28MWB//Si/6-311+G(d) level. All energies are in eV. Page 21

Table S4. The NPA charges (e) on the parent Si atoms, double-Gd atoms and total charges for the most stable isomers. Page 22

Table S5. The valence electron configuration of each one of the two Gd atoms, magnetic moments (μ_B) of the Gd-4f orbital, total magnetic moments of the two Gd atoms, and total magnetic moments for the most stable isomers. Page 23

Figure S1. (a) Photoelectron spectrum of $GdSi_4^-$ measured at 266 nm (4.66 eV), the spectrum is taken from Ref. 20. (b) Simulated photoelectron spectra (PES) from the lowest-energy structures for $GdSi_4^-$ clusters at the PBE0/Gd/ECP28MWB//Si/6-311+G(d) level, together with B3LYP, PBE, TPSSh and BPW91 functionals for comparison. Each VDE was fitted with a full width at half-maximum (FWHM) of 0.20 eV to yield the simulated PES spectra. Page 24

Figure S2. The various geometrical structures of $Gd_2Si_n^-$ (n =3-7) with relative energy, symmetry and electron state at the PBE0/Gd/ECP28MWB//Si/6-311+G(d) level. The gray and light green balls represent silicon and gadolinium atoms, respectively. Page 25

Figure S3. The various geometrical structures of $Gd_2Si_n^-$ (n =8-12) with relative energy, symmetry and electron state at the PBE0/Gd/ECP28MWB//Si/6-311+G(d) level. The gray and light green balls represent silicon and gadolinium atoms, respectively. Page 26

Figure S4. The growth behavior of $Gd_2Si_n^-$ (n =5-12) clusters. Page 27

Figure S5. The simulated photoelectron spectra (PESs) for the assigned major clusters within 0.3eV at the PBE0/Gd/ECP28MWB//Si/6-311+G(d) level. The simulated PESs exhibit a full width at half maximum (FWHM) of 0.20 eV. Page 28

Figure S6. The average binding energies of $Gd_2Si_n^-$ (n =3-12) and Si_{n+2}^- at PBE0/Gd/ECP 28MWB//Si/6-311+G(d) level. Page 29
Figure S7. The spin-density (ρ_{alpha}–ρ_{beta}) isosurfaces of lowest-lying isomers of $Gd_2Si_n^-$ (n=3-12). The isosurface is set to ±0.02. The red and blue isosurfaces show that the spin density has positive and negative values, respectively. Page 30

Table S1. Comparison of ADEs and VDEs using different methods for $GdSi_4^-$

Species	Methods	ADEs	VDEs
$GdSi_4^-$	PBE0	**2.17**	**2.51**
	PBE	2.00	2.03
	B3LYP	2.29	2.43
	BPW91	1.88	1.91
	TPSSh	1.94	2.17
	Expt.[a]	2.15 ± 0.05	2.50 ± 0.1

[a] Reference 19.

Table S2. Cartesian coordinates for the top low-lying isomers of $Gd_2Si_n^-$ (n =3-12) at the PBE0/Gd/ECP28MWB//Si/6-311+G(d) level.

**3-A**			
Atom	X	Y	Z
Si	0.00000000	1.50067200	0.00000000
Si	1.29962000	-0.75033600	0.00000000
Si	-1.29962000	-0.75033600	0.00000000
Gd	0.00000000	0.00000000	2.28002500
Gd	0.00000000	0.00000000	-2.28002500
**3-B**			
Atom	X	Y	Z
Si	0.00000000	1.98529400	0.91936600
Si	0.00000000	0.00000000	1.98112000
Si	0.00000000	-1.98529400	0.91936600
Gd	-1.66473400	0.00000000	-0.41779600
Gd	1.66473400	0.00000000	-0.41779600
**3-C**			
Atom	X	Y	Z
Si	0.00000000	0.00000000	3.26206900
Si	0.00000000	1.21986600	1.31267000
Si	0.00000000	-1.21986600	1.31267000
Gd	1.76708900	0.00000000	-0.64393500
Gd	-1.76708900	0.00000000	-0.64393500
**3-D**			
Atom	X	Y	Z
Si	-0.14060300	2.19105300	1.13544200
Si	1.56671600	0.95666600	0.00000000
Si	-0.14060300	2.19105300	-1.13544200
Gd	-0.14060300	-0.58392800	-1.78572000
Gd	-0.14060300	-0.58392800	1.78572000
**3-E**			
Atom	X	Y	Z
Gd	2.68852000	-0.02804900	0.00001700
Gd	-2.37910700	-0.42695400	0.00002200
Si	0.12944100	1.09034000	-0.00002500
Si	-1.81856200	2.25094900	-0.00003400
Si	0.27466300	-1.26127900	-0.00011800
**3-F**			
Atom	X	Y	Z
Gd	-1.64068500	0.54268600	-0.07703900
Si	-1.31234300	-2.10489200	0.41372400
Si	0.76740600	2.14346200	0.26986900
Gd	1.14084200	-0.87972300	-0.09012600
Si	2.82992900	1.50217400	0.08059200
**4-A**			
Atom	X	Y	Z

Gd	2.54599900	-0.09352200	-0.00005700
Gd	-2.41284100	-0.29390400	-0.00005100
Si	0.08078000	-1.56126800	0.00036200
Si	0.15618300	0.56623700	1.27183100
Si	-1.00175800	2.20010000	0.00007100
Si	0.15607300	0.56601800	-1.27176900
**4-B**			
Atom	X	Y	Z
Si	0.00000000	1.17136500	2.00145200
Si	0.00000000	-1.17136500	2.00145200
Si	-1.25603800	0.00000000	0.22222000
Si	1.25603800	0.00000000	0.22222000
Gd	0.00000000	2.45571100	-0.48642800
Gd	0.00000000	-2.45571100	-0.48642800
**4-C**			
Atom	X	Y	Z
Gd	-2.23355800	0.00000000	0.00000000
Gd	2.23355800	0.00000000	0.00000000
Si	0.00000000	0.00000000	1.59948900
Si	0.00000000	1.87042200	0.00000000
Si	0.00000000	-1.87042200	0.00000000
Si	0.00000000	0.00000000	-1.59948900
**4-D**			
Atom	X	Y	Z
Gd	-1.84204500	-0.17921100	-0.24741000
Gd	1.52743800	-0.76918000	-0.24676300
Si	1.93529500	1.96662400	0.28286600
Si	-0.16891600	1.51749500	1.20581700
Si	-0.34978300	-0.79082200	1.94064100
Si	0.02160500	1.64220500	-1.17024700
**4-E**			
Atom	X	Y	Z
Gd	-1.62465300	-0.87173700	-0.00001400
Gd	1.98728100	-0.41780400	-0.00000500
Si	0.60461300	2.23575400	-0.00006000
Si	-1.75149300	2.06931800	0.00023500
Si	-0.25553400	0.79509300	-1.65282400
Si	-0.25531100	0.79488100	1.65273800
**4-F**			
Atom	X	Y	Z
Gd	2.39466500	-0.00000500	-0.00001600
Gd	-2.39466400	-0.00000600	-0.00001700
Si	0.00099700	-1.68423000	0.01714700
Si	-0.00099700	0.01711800	1.68427300
Si	-0.00099000	-0.01709200	-1.68420300
Si	0.00098600	1.68425400	-0.01706300
**5-A**			
Atom	X	Y	Z

Si	0.00000000	1.22762800	-2.10534600
Si	1.22955600	0.00000000	-0.40527700
Si	0.00000000	0.00000000	1.64809200
Si	0.00000000	-1.22762800	-2.10534600
Si	-1.22955600	0.00000000	-0.40527700
Gd	0.00000000	2.60376600	0.36893900
Gd	0.00000000	-2.60376600	0.36893900
**5-B**			
Atom	X	Y	Z
Si	0.00000000	-1.93040936	-1.91452363
Si	1.23709055	0.00000000	-1.14749367
Si	0.00000000	1.93040936	-1.91452363
Si	0.00000000	0.00000000	-3.33097399
Si	-1.23709055	0.00000000	-1.14749367
Gd	0.00000000	1.58027968	1.03409254
Gd	0.00000000	-1.58027968	1.03409254
**5-C**			
Atom	X	Y	Z
Gd	-2.54867800	-0.21830800	-0.00044000
Gd	1.82369300	-1.02340700	-0.00254400
Si	-0.19032000	0.24692000	1.97588200
Si	-0.16791700	1.86064200	0.00502800
Si	1.93308200	1.65154300	1.46334500
Si	1.93009400	1.66022100	-1.45702300
Si	-0.19071900	0.25708300	-1.97359300
**5-D**			
Atom	X	Y	Z
Si	0.00000000	-2.03209104	0.87227028
Si	0.00000000	-1.31835094	-1.41919107
Si	0.00000000	0.00000000	1.97622714
Si	0.00000000	1.31835094	-1.41919107
Si	0.00000000	2.03209104	0.87227028
Gd	2.10611220	0.00000000	-0.09569654
Gd	-2.10611220	0.00000000	-0.09569654
**5-E**			
Atom	X	Y	Z
Gd	-2.17328300	-0.47584300	0.00039800
Gd	2.40893100	-0.06054800	-0.00019900
Si	0.09918100	1.27195000	1.19488000
Si	0.20335300	-1.18425400	-1.30702100
Si	0.10069900	1.26552100	-1.19665100
Si	-1.68442500	2.27582800	-0.00380600
Si	0.20394500	-1.17696800	1.31168800
**5-F**			
Atom	X	Y	Z
Gd	-0.84467000	1.30203400	-0.00011300
Gd	2.20799200	-0.12190100	0.00001200
Si	-2.03404900	-0.81176800	2.16055700
Si	-2.03404000	-0.81219000	-2.16042600

Si	0.07926900	-1.20829600	-1.26450900
Si	-2.32277200	-1.35457700	0.00012100
Si	0.07926400	-1.20806100	1.26472300
**6-A**			
Atom	X	Y	Z
Gd	0.00000000	2.23494500	-0.42401400
Gd	0.00000000	-2.23494500	-0.42401400
Si	1.35345700	0.00000000	0.99247600
Si	-1.28346100	0.00000000	-1.51617400
Si	-1.35345700	0.00000000	0.99247600
Si	0.00000000	-1.26976400	2.46204500
Si	0.00000000	1.26976400	2.46204500
Si	1.28346100	0.00000000	-1.51617400
**6-B**			
Atom	X	Y	Z
Si	1.70432000	1.94403100	0.54255900
Si	-1.70429600	1.94404000	0.54255300
Si	-0.00000200	-1.85645000	1.33968600
Si	0.00001800	3.47259100	-0.26969900
Si	0.00001200	0.51253500	1.37247200
Si	0.00001300	1.21420600	-1.08684700
Gd	2.01850200	-0.79089500	-0.26695600
Gd	-2.01851600	-0.79087600	-0.26695200
**6-C**			
Atom	X	Y	Z
Gd	-2.73674300	-0.27755900	0.08534300
Gd	2.41703800	-0.65284300	-0.14550100
Si	-0.32209100	-1.69658700	0.46113400
Si	1.99195900	1.59103400	1.51355100
Si	-0.21875800	-0.10144400	-1.40545800
Si	-0.07105300	0.54788300	1.08901500
Si	-1.15672200	2.00519300	-0.53931100
Si	1.23817300	1.90719100	-0.84391900
**6-D**			
Atom	X	Y	Z
Gd	-2.52737900	-0.12972500	-0.30267800
Gd	2.61751500	-0.33054000	-0.17358700
Si	-1.03686200	2.25628800	0.34938000
Si	0.28545200	0.93645300	-1.18789800
Si	1.33279300	2.09755500	0.75143400
Si	0.09077300	-1.59227900	-0.54498700
Si	-0.06049900	0.25509500	1.25127100
Si	-1.02370600	-1.84904100	1.55800900
**6-E**			
Atom	X	Y	Z
Gd	2.03582900	-0.42487400	-0.17863200
Gd	-2.24762200	-0.02543800	-0.09825300
Si	-0.19091000	1.63760300	1.23274900
Si	-0.24884500	-1.84439400	0.71747700

Si	-0.18868200	1.38441100	-1.27826700
Si	1.55690000	2.35967100	-0.08976600
Si	0.25271900	-0.38877900	2.38559700
Si	-0.21298100	-1.08994200	-1.70202700
**6-F**			
Atom	X	Y	Z
Si	0.00000000	1.60966400	0.92735800
Si	0.00000000	1.28020100	-1.54167200
Si	0.00000000	-1.60966400	0.92735800
Si	1.19659500	0.00000000	2.31540400
Si	0.00000000	-1.28020100	-1.54167200
Si	-1.19659500	0.00000000	2.31540400
Gd	-2.12615800	0.00000000	-0.37211300
Gd	2.12615800	0.00000000	-0.37211300
**7-A**			
Atom	X	Y	Z
Gd	2.84256500	-0.32493300	0.00014100
Gd	-2.35641400	-0.77535000	0.00008800
Si	0.41436400	-1.76533500	0.00099100
Si	0.30784800	0.21835100	1.36438700
Si	-1.06177100	2.03151300	-0.00023900
Si	1.31299800	2.02553500	-0.00068100
Si	-1.75222100	1.15217000	2.06166200
Si	-1.75173900	1.15087100	-2.06217500
Si	0.30812000	0.21675700	-1.36499100
**7-B**			
Atom	X	Y	Z
Gd	2.42889300	-0.37531500	0.07748300
Gd	-2.22931300	-0.31037400	0.41918900
Si	1.28572100	2.40869400	-0.18236800
Si	0.17456000	-1.48774600	-1.14393300
Si	0.04185800	0.93093100	-1.51060700
Si	-1.12514400	2.39438400	0.16013300
Si	-1.56803000	-0.50217900	-2.35858400
Si	0.09115400	-1.54592600	1.32357000
Si	0.18751500	0.93642100	1.44129100
**7-C**			
Atom	X	Y	Z
Si	0.00000100	3.46238100	0.54487000
Si	-1.72093500	2.04775300	-0.40535400
Si	-0.00000500	0.71949500	-1.43119600
Si	0.00000100	-1.32703600	1.52629000
Si	0.00000100	-1.71222100	-1.10402100
Si	0.00001100	1.08616000	1.02384900
Si	1.72093900	2.04775200	-0.40537800
Gd	2.31911200	-0.69172000	0.02744800
Gd	-2.31911500	-0.69171800	0.02744500

7-D			
Atom	X	Y	Z
Gd	-2.52164800	-0.49002700	0.00000000
Gd	2.41018400	0.18538800	0.00000000
Si	1.69343800	-2.50341400	0.00000000
Si	-0.11799600	0.97030900	1.31892200
Si	-0.11799600	-1.52356900	-1.22499200
Si	-0.11799600	0.97030900	-1.31892200
Si	-1.57402700	2.30075800	0.00000000
Si	0.86212300	2.70181300	0.00000000
Si	-0.11799600	-1.52356900	1.22499200
**7-E**			
Atom	X	Y	Z
Si	0.24093400	0.00000000	2.39006100
Si	-0.33221500	1.76720200	0.94169900
Si	-0.31372100	-1.25419400	-1.47297400
Si	-0.31372600	1.25419400	-1.47297400
Si	1.27822000	-2.76053500	-0.40142600
Si	1.27820800	2.76053900	-0.40142800
Si	-0.33221200	-1.76720100	0.94169500
Gd	-2.30120600	-0.00000300	-0.06073500
Gd	1.97188000	0.00000200	-0.05403300
**7-F**			
Atom	X	Y	Z
Gd	-2.03956600	-0.34587700	-0.07135400
Gd	2.01083000	-0.31153000	0.00022800
Si	-1.28971300	2.69487900	0.02939600
Si	-0.03310700	1.17075000	1.30065900
Si	1.32502900	2.68956800	0.05955700
Si	0.22675600	-2.47277600	0.11682700
Si	0.01889800	1.20483300	-1.27815900
Si	0.10970300	-1.15740600	-1.92242800
Si	-0.22619700	-1.12456000	2.01929500
**8-A**			
Atom	X	Y	Z
Gd	2.44443300	-0.65315500	0.02200900
Gd	-2.61426400	-0.53819100	-0.11919000
Si	-1.15918900	1.24451200	1.72629000
Si	0.70860100	2.09280500	0.05214200
Si	1.89160500	1.45346900	-1.84030900
Si	-1.55260500	2.09574200	-0.63986600
Si	1.20965900	0.93795600	2.15825900
Si	-0.09171800	-0.92524600	1.41333100
Si	-0.14003800	0.38316400	-1.64001800
Si	-0.08994200	-1.83625200	-0.78557400
**8-B**			
Atom	X	Y	Z

Gd	2.58918900	-0.59564600	0.00061000
Gd	-2.60104100	-0.71470500	-0.00124900
Si	0.03971600	-1.25167700	-1.21331100
Si	-0.82908000	0.94378800	-1.66200600
Si	1.40178100	0.99759600	-1.98055500
Si	1.39499800	0.99108100	1.98146300
Si	-0.83827700	0.95044700	1.66560300
Si	0.70255100	2.34102300	0.00078500
Si	0.03795800	-1.24428600	1.21625800
Si	-1.85546700	2.26220500	-0.00532000
**8-C**			
Atom	X	Y	Z
Si	1.61916000	-1.22303000	1.27152300
Si	-0.55985600	-1.47536800	0.00000000
Si	0.05360500	1.16262000	0.00000000
Si	1.61916000	1.15892200	-1.79577900
Si	1.61916000	1.15892200	1.79577900
Si	1.61916000	-1.22303000	-1.27152300
Si	3.14532200	0.51931800	0.00000000
Si	-2.12147400	0.47692700	0.00000000
Gd	-0.76499500	-0.06073400	-2.47878400
Gd	-0.76499500	-0.06073400	2.47878400
**8-D**			
Atom	X	Y	Z
Si	-0.00003100	3.65553200	-0.62602200
Si	1.52850300	2.25546300	0.59585400
Si	-0.00000700	1.27225800	-0.99237500
Si	0.00000100	0.72352800	1.61645000
Si	-1.52852800	2.25543600	0.59586200
Si	0.00000500	-0.82709600	-2.08061800
Si	0.00000000	-1.51594400	1.97211200
Si	0.00001500	-2.50185300	-0.17367200
Gd	2.11279100	-0.58157200	-0.09926700
Gd	-2.11278200	-0.58159300	-0.09926900
**8-E**			
Atom	X	Y	Z
Si	-2.20587400	1.02105600	1.23856400
Si	-0.20632100	1.73575200	0.00016300
Si	0.07716200	0.38720400	-2.05320600
Si	1.01273100	-1.66873000	-1.26837500
Si	-2.20556500	1.02116100	-1.23871300
Si	1.01257100	-1.66868500	1.26872400
Si	-2.11223800	3.12687700	-0.00004700
Si	0.07665800	0.38705800	2.05351400
Gd	2.45202300	0.47369000	-0.00000300
Gd	-1.45651900	-1.42343600	-0.00013300
**8-F**			
Atom	X	Y	Z

Gd	2.45830300	-0.86321000	-0.36933500
Gd	-2.83251500	-0.47454600	-0.23444500
Si	-0.20112800	-1.68801800	0.49244700
Si	1.31966500	1.53772400	-1.28926200
Si	-1.03831800	1.77592900	-0.18583700
Si	0.56646100	3.53178900	-0.01754500
Si	-0.32176200	-0.26068600	-1.48094900
Si	1.16817200	1.53121400	1.09895200
Si	1.18511000	-0.53161900	2.31333000
Si	-0.96751800	0.21912200	1.82899800
**9-A**			
Atom	X	Y	Z
Si	1.56734500	0.76501600	1.33057400
Si	1.01325000	-1.27014800	0.00000000
Si	3.12338800	1.97099900	0.00000000
Si	-1.16103500	0.54395900	0.00000000
Si	-0.48988700	1.96310300	-1.78483700
Si	-0.48988700	1.96310300	1.78483700
Si	1.56734500	0.76501600	-1.33057400
Si	0.88260700	2.93205000	0.00000000
Si	-1.53416000	-1.72647300	0.00000000
Gd	-0.48988700	-0.86478700	2.55308100
Gd	-0.48988700	-0.86478700	-2.55308100
**9-B**			
Atom	X	Y	Z
Gd	2.54737500	-1.20175000	-0.00001100
Gd	-2.49317700	-0.97714400	-0.45994100
Si	-1.92303300	-0.51042600	2.31233500
Si	0.13691600	3.13957400	1.38251400
Si	2.27086200	1.27597800	-1.31946600
Si	-1.31495000	1.34139400	0.99858300
Si	0.14385400	-0.87868000	1.29985100
Si	0.40895000	2.79322400	-1.00754700
Si	0.19976200	0.00642800	-1.17931100
Si	1.45407700	1.24486100	1.02695000
Si	-1.62420200	1.54830200	-1.41127300
**9-C**			
Atom	X	Y	Z
Gd	2.67398000	-0.58286100	-0.34318700
Gd	-2.60571400	-0.50141800	-0.50587400
Si	0.07924000	-1.05021300	-1.81086100
Si	0.03521000	1.27196300	-0.97962400
Si	0.99744600	-0.62108300	2.21585100
Si	-1.25001600	1.52102800	1.53750300
Si	-2.03169000	2.57320000	-0.65183200
Si	2.19710300	2.44017500	-0.72386500
Si	1.19140600	1.82183900	1.50541000
Si	-1.47053800	-0.86976300	2.34033700

Si	-0.06023500	-2.13044400	0.44850500
**9-D**			
Atom	X	Y	Z
Gd	-2.57892200	-0.28319000	-0.60163200
Gd	2.80560600	-0.75123800	-0.29109100
Si	-0.82896100	1.76665200	-1.70081400
Si	-1.25153900	0.06911500	2.24374000
Si	1.03696300	0.54355700	1.65057300
Si	1.06741800	2.85160400	1.31969700
Si	-1.91678800	-2.11399700	1.51492900
Si	0.24113200	-0.39345000	-1.49496000
Si	1.42924200	1.70090400	-0.82742800
Si	0.17659200	-1.58260700	0.62334300
Si	-0.99032600	1.88704100	0.75194100
**9-E**			
Atom	X	Y	Z
Gd	-2.66920200	-0.19801500	-0.22366100
Gd	2.65382500	-0.13033900	-0.36179200
Si	1.20721300	-2.53080000	0.74462700
Si	-0.97895700	1.67142700	1.68018400
Si	0.12209000	0.76708300	-1.52327600
Si	-1.44969600	2.35484100	-0.62761200
Si	-0.03951900	-1.57329800	-1.18855500
Si	0.00917600	-0.47699300	0.98847500
Si	0.91453300	2.55393500	-0.10865000
Si	1.46546900	1.24375200	1.86152200
Si	-1.18001500	-2.50889700	0.84964400
**9-F**			
Atom	X	Y	Z
Gd	-2.01032700	-0.25349900	-0.62605900
Gd	1.41357400	-0.66170800	-1.03720700
Si	-0.44507300	-1.00219900	1.78576400
Si	-0.39425000	-2.55508300	0.10805300
Si	-0.23319400	1.52538300	0.99180200
Si	-0.02125300	1.83587500	-1.34608800
Si	-2.05170400	0.65409900	2.21353200
Si	0.35261800	3.68890100	0.21192400
Si	1.99844600	1.94922200	0.06027100
Si	1.59269100	-2.14777100	1.69029500
Si	1.92973500	0.23537800	1.88795200
**10-A**			
Atom	X	Y	Z
Si	0.00000200	3.48633600	-0.08261100
Si	-1.91446900	2.15331400	0.51393800
Si	1.91447100	2.15331300	0.51394100
Si	-1.16636600	0.37983800	1.90800500
Si	1.16635200	0.37984000	1.90800000
Si	-0.00000100	-0.90575100	-1.75735100

Si	2.06622100	-1.75159700	1.73946400
Si	-2.06622000	-1.75160600	1.73945500
Si	-0.00000100	1.04471200	-0.45733500
Si	0.00000400	-1.62158200	0.60557100
Gd	2.66262300	-0.39012100	-0.72527400
Gd	-2.66262100	-0.39012000	-0.72527400
**10-B**			
Atom	X	Y	Z
Gd	-2.45686400	-0.93779400	-0.27577800
Gd	2.57243000	-1.04860400	0.19986500
Si	1.44707700	1.54609800	0.84685300
Si	-1.60489200	1.39840400	-1.60284400
Si	0.34747200	2.79485800	-1.16645800
Si	0.07763500	-0.57615500	1.39496600
Si	0.16922600	-0.19411300	-1.42195400
Si	0.14704300	3.51673600	1.11649700
Si	-1.28546400	1.63096600	0.80679500
Si	0.04650200	-2.30085900	-0.47319700
Si	2.10756600	1.24831500	-1.50115700
Si	-1.98046600	0.01642800	2.34752700
**10-C**			
Atom	X	Y	Z
Si	-1.36611400	1.15598700	1.27174800
Si	-0.00000100	-1.16499000	1.40608200
Si	0.00000100	2.77613500	2.36503400
Si	-0.00000100	-0.14933700	-1.13443700
Si	1.74626000	1.44428500	-1.19871900
Si	-1.74625700	1.44428600	-1.19871800
Si	1.36611800	1.15598700	1.27174900
Si	0.00000200	2.99583100	0.04572800
Si	-0.00000300	-2.41207800	-0.84632300
Si	0.00000300	2.84713600	-2.26298300
Gd	2.46548800	-1.10395000	0.03071700
Gd	-2.46549000	-1.10394700	0.03071700
**10-D**			
Atom	X	Y	Z
Gd	-2.28937900	-1.49138900	-0.00086400
Gd	2.75672200	-1.02825700	0.00009800
Si	0.02141500	2.76683000	-2.35020800
Si	0.16070300	-1.02983300	-1.26516800
Si	0.10148700	2.70384500	-0.00001300
Si	0.16023100	-1.03409700	1.26619500
Si	-3.23051600	1.12150600	0.00236100
Si	-1.20186500	1.00952200	1.30438600
Si	1.51583100	1.10022000	1.34218900
Si	-1.20445900	1.01097200	-1.30383200
Si	1.51911900	1.10177500	-1.34285600
Si	0.02162900	2.76764100	2.35044700

10-E			
Atom	X	Y	Z
Gd	-0.57224500	2.14988700	0.55778300
Gd	-1.99953500	-0.64266300	-0.82793300
Si	2.89791400	0.93417300	-2.09018100
Si	2.26993500	1.30418300	0.18486400
Si	0.55326600	0.46903000	-1.77889700
Si	0.57050400	-2.02329200	-1.48519400
Si	2.73620800	-1.14504600	-0.94421700
Si	-0.56030000	-2.71299000	0.78224600
Si	2.94215300	-0.70703600	1.39156300
Si	-1.16038000	-0.56261000	2.00378100
Si	0.86109800	-1.86930100	2.62424700
Si	0.64631000	-0.57727500	0.54675600
**10-F**			
Atom	X	Y	Z
Gd	-2.26371600	0.63904700	-0.68380500
Gd	2.93262700	-0.54583400	-0.38598600
Si	-0.46412600	0.97327200	1.74091100
Si	-3.59846300	-1.56757700	0.49992200
Si	1.73181900	0.14633900	2.19942800
Si	1.98430400	2.01331700	0.43821000
Si	0.27149100	-1.19093300	0.85536900
Si	-0.34357500	2.81607200	0.16210900
Si	-1.84619900	-0.95762900	1.97994700
Si	-1.53274600	-2.47345400	-0.29789100
Si	0.49651000	1.06525700	-1.17459800
Si	0.24310400	-1.25077800	-1.51293000
**11-A**			
Atom	X	Y	Z
Si	3.06059400	-1.58626800	0.00000000
Si	1.13527900	-1.05925000	1.30716200
Si	-2.43639400	0.67954100	0.00000000
Si	1.13527900	1.31433700	-1.97646100
Si	1.13527900	-1.05925000	-1.30716200
Si	1.13527900	1.31433700	1.97646100
Si	-1.41998500	-1.62790800	0.00000000
Si	2.14318500	2.47482300	0.00000000
Si	-0.06919700	0.86453400	0.00000000
Gd	-1.37188700	-0.27671600	-2.44770900
Gd	-1.37188700	-0.27671600	2.44770900
Si	3.36182700	0.60753700	-1.22903500
Si	3.36182700	0.60753700	1.22903500
**11-B**			
Atom	X	Y	Z
Si	1.93782000	-0.14430400	2.39629000
Si	-1.33012200	1.51783900	0.83379200
Si	0.00032200	-0.90611100	1.32565300

Si	-0.00011400	3.43749300	1.11444000
Si	0.00030200	0.01561900	-1.35918500
Si	1.89073300	1.46459500	-1.54935100
Si	-1.89093600	1.46411600	-1.54854200
Si	1.32889900	1.51738000	0.83297000
Si	-0.00043300	3.00965800	-1.27952100
Si	0.00002600	-2.24259300	-0.66269300
Si	-1.93772200	-0.14558200	2.39635600
Gd	2.57486600	-0.98296200	-0.27332700
Gd	-2.57459800	-0.98318700	-0.27359300
**11-C**			
Atom	X	Y	Z
Gd	-2.52858100	-1.09908100	0.20006600
Gd	2.70349500	-0.80959600	-0.55913000
Si	-2.07190200	1.42592000	1.46648100
Si	1.26059800	1.65438200	-0.85496300
Si	-0.02066400	2.72437200	1.18968500
Si	0.07701600	-0.53041000	-1.49895800
Si	1.94170900	1.41756700	1.54239900
Si	-0.17445000	0.05748000	1.43373300
Si	-1.49404400	1.56012300	-0.94945600
Si	0.15859900	-2.06438800	0.50367100
Si	-2.08172200	-0.14440000	-2.42621700
Si	1.75126900	-0.91414000	2.30088800
Si	-0.14601600	3.53887100	-1.06582900
**11-D**			
Atom	X	Y	Z
Gd	2.29464300	-1.50538200	0.06594700
Gd	-2.88241900	-0.81663700	0.00623800
Si	0.09900300	2.99526200	2.21647000
Si	-0.19193500	-0.38412900	1.08623200
Si	0.06471300	2.90375200	-0.09128200
Si	-0.16335000	-1.04591500	-1.43701100
Si	3.48667000	1.08786500	-0.11991600
Si	1.32344100	0.96515800	-1.22002900
Si	-1.47018500	1.19680200	-1.32478900
Si	1.52082600	1.28881200	1.24968100
Si	-1.67339000	1.51874900	1.24681700
Si	0.14999400	2.64894100	-2.40924100
Si	-0.45881000	-2.56035500	0.47308000
**11-E**			
Atom	X	Y	Z
Si	-0.14078100	-1.38294100	-0.00004700
Si	0.54991600	0.53606000	-1.49884900
Si	2.21640500	1.86895000	0.00007000
Si	-1.81991400	1.62734300	1.27138500
Si	0.54990600	0.53595700	1.49888800
Si	-1.81990500	1.62742900	-1.27128900

Si	-0.03704100	2.53833600	0.00008600
Si	-1.31021000	-0.68746600	2.06915500
Si	-1.31019500	-0.68732600	-2.06921100
Si	2.76504200	0.91875000	2.07211300
Si	2.76505700	0.91888900	-2.07203300
Gd	-3.25721700	-0.56787100	-0.00003100
Gd	2.73040600	-1.14143700	-0.00002800
**11-F**			
Atom	X	Y	Z
Si	-1.01815200	-0.89651400	1.92465300
Si	-1.97239500	-2.73275700	0.77719100
Si	-0.04751000	-1.71376500	-0.16323900
Si	1.28201400	-1.25602900	1.82840800
Si	-1.75948700	1.38934400	1.66187900
Si	0.38388700	2.43640000	1.43833100
Si	2.26165700	1.01539000	1.42775700
Si	-0.03586400	0.06356800	-1.84044300
Si	1.47836700	1.69011900	-0.77334700
Si	0.42570400	3.84346000	-0.51119800
Si	-1.03795100	1.93943700	-0.60289300
Gd	2.79818700	-0.93174700	-0.52697800
Gd	-2.78949600	-0.33233300	-0.60332500
**12-A**			
Atom	X	Y	Z
Si	2.08320600	2.90524900	0.00000000
Si	1.29717700	1.01660100	1.22229100
Si	-0.96179800	1.56072400	-2.04062800
Si	1.29717700	1.01660100	-1.22229100
Si	-0.96179800	1.56072400	2.04062800
Si	2.22692400	-1.02454600	0.00000000
Si	-2.04092700	2.55330900	0.00000000
Si	-0.94732900	0.34346400	0.00000000
Si	-0.03671200	3.67947900	-1.18765300
Si	-0.03671200	3.67947900	1.18765300
Si	0.76698800	-2.87408200	0.00000000
Si	-1.65507200	-1.93169100	0.00000000
Gd	-0.11277900	-1.36558100	-2.27987300
Gd	-0.11277900	-1.36558100	2.27987300
**12-B**			
Atom	X	Y	Z
Si	2.41299700	-0.56442000	2.17786900
Si	-1.04674000	1.40377500	1.07182300
Si	0.26436000	-0.96252200	1.37212800
Si	0.38282200	3.20315300	1.69126300
Si	0.01109900	0.33752800	-1.19590000
Si	2.01956000	1.65907900	-1.37296400
Si	-1.66912600	2.04362400	-1.23580700
Si	1.65464100	1.36443400	0.98958400

Si	0.31154900	3.27688300	-0.74651800
Si	-0.11095900	-2.03724100	-0.82790500
Si	-1.59273400	-2.49664700	1.12910700
Si	-1.80302700	-0.42821200	2.43908300
Gd	2.58111900	-1.02844300	-0.60974500
Gd	-2.76365300	-0.45893300	-0.59157900
**12-C**			
Atom	X	Y	Z
Gd	-2.58589000	-1.40654200	-0.07739700
Gd	2.65514100	-1.30347800	0.03986900
Si	-0.21601200	3.04691600	-2.19378500
Si	-0.02475300	-0.39860100	-1.08939900
Si	-0.06781900	2.87881600	0.09932200
Si	0.06942300	-0.95580000	1.37460100
Si	-3.59396500	1.27672000	0.31817600
Si	-1.47221400	1.12274500	1.33150300
Si	1.48552500	1.14168100	1.11484100
Si	-1.72271100	1.40570200	-1.12470700
Si	1.47205200	1.44532500	-1.36083700
Si	0.09682200	2.61409300	2.40582900
Si	0.12390700	-2.60796200	-0.52190100
Si	3.53316900	1.41903400	-0.18208600
**12-D**			
Atom	X	Y	Z
Gd	-1.82825500	-0.34546000	-1.38644200
Gd	0.60879300	-2.27984900	0.74575900
Si	-2.82298300	1.29197800	0.86035500
Si	3.46005400	-0.53599100	-1.29997200
Si	0.39289100	1.61935800	-2.44049000
Si	1.10567300	-0.46207300	-1.41801300
Si	2.62818200	2.42773500	1.11153500
Si	-1.46149300	1.36034500	2.85206700
Si	-1.83343600	-0.87496900	1.55736200
Si	2.43825000	1.68236800	-1.15747500
Si	2.37960200	0.06230100	0.86392500
Si	-0.85883700	2.21623200	-0.46244400
Si	0.18593600	2.73888600	1.59212900
Si	-0.03915600	0.47524100	0.86985700
**12-E**			
Atom	X	Y	Z
Si	2.40646900	-0.19091200	-1.88641300
Si	0.64222900	-1.51753600	-1.05762500
Si	-2.68574900	0.11056200	0.63575100
Si	1.02850500	1.93376600	1.32611100
Si	0.95250700	1.50835400	-1.08314700
Si	0.89397700	-1.92571400	1.45985000
Si	-1.39820700	0.06076800	-1.43973400
Si	2.37869000	0.11259100	2.29643100

Si	-0.38335500	0.05815900	0.90851300
Si	3.32135700	1.09406700	0.23559000
Si	2.88551400	-1.33032500	0.33215300
Si	4.64301700	-0.62848900	-1.00843200
Gd	-1.78056300	-2.51657300	-0.04609800
Gd	-1.43177100	2.67291600	-0.11119400
**12-F**			
Atom	X	Y	Z
Gd	-2.69485000	0.42775300	-0.57574100
Gd	2.78558600	-0.24436400	-0.81982700
Si	1.11386800	-1.25719900	1.57075400
Si	-0.93334700	-0.15207600	1.88140300
Si	0.61141200	-2.87168800	-0.20524100
Si	-1.66951300	-2.39275100	0.44723800
Si	3.11456600	0.00758500	1.93516200
Si	-3.00273200	-1.16285200	1.94993200
Si	-1.16916300	3.07101400	-0.61365700
Si	0.02572200	-0.37832800	-0.43008900
Si	2.01881000	2.08620700	1.25840900
Si	0.91581400	1.98364900	-0.89865600
Si	-1.12769200	-1.87537700	-1.80151100
Si	-0.31253800	2.10346300	1.28600000

Table S3. Various structures with type the spin multiplicity (SM), the symmetry (Sym), the relative energy (ΔE), the first adiabatic detachment energy (ADE) and first vertical detachment energy (VDE) for the low-lying isomers of $Gd_2Si_n^-$ (n=3-12) at the PBE0/Gd/ECP28MWB//Si/6-311+G(d) level. All energies are in eV.

Cluster	Isomer	SM	Sym	ΔE	ADE	VDE
$Gd_2Si_3^-$	A	16	D_{3h}	0.00	1.20	1.37
	B	16	C_{2v}	0.31	1.46	1.50
	C	16	C_{2V}	0.70	1.19	1.23
	D	16	C_s	0.72	1.23	1.28
$Gd_2Si_4^-$	A	16	C_1	0.00	1.11	1.36
	B	18	C_{2V}	0.22	0.43	0.51
	C	18	D_{2h}	0.34	0.96	1.53
	D	16	C_1	0.42	1.50	1.84
$Gd_2Si_5^-$	A	16	C_{2v}	0.00	0.78	0.84
	B	16	C_{2v}	0.30	1.31	1.43
	C	16	C_1	0.65	1.86	2.09
	D	16	C_{2V}	0.96	6.90	7.10
$Gd_2Si_6^-$	A	16	C_{2v}	0.00	1.95	2.09
	B	16	C_s	0.20	1.83	1.94

	C	16	C_1	0.34	1.50	1.70
	D	16	C_1	0.41	1.63	1.76
$Gd_2Si_7^-$	A	16	C_s	0.00	2.16	2.20
	B	16	C_1	0.10	2.01	2.38
	C	16	C_s	0.13	1.99	2.33
	D	16	C_s	0.16	2.26	2.42
$Gd_2Si_8^-$	A	16	C_1	0.00	1.77	2.19
	B	16	C_1	0.40	1.56	2.05
	C	16	C_s	0.40	2.13	2.53
	D	16	C_s	0.49	2.19	2.59
$Gd_2Si_9^-$	A	18	C_s	0.00	1.75	1.86
	B	16	C_1	0.20	1.62	1.82
	C	18	C_1	0.43	1.87	1.93
	D	18	C_1	0.48	1.94	2.12
$Gd_2Si_{10}^-$	A	16	C_s	0.00	2.26	2.31
	B	16	C_1	0.02	2.02	2.16
	C	16	C_s	0.34	1.91	2.17
	D	16	C_1	0.83	2.09	2.28
$Gd_2Si_{11}^-$	A	16	C_s	0.00	2.31	2.54
	B	16	C_s	0.06	2.08	2.37
	C	16	C_1	0.42	2.27	2.44
	D	16	C_1	0.55	2.59	2.69
$Gd_2Si_{12}^-$	A	16	C_s	0.00	2.53	2.85
	B	16	C_1	0.005	2.25	2.51
	C	16	C_1	0.43	2.51	2.37
	D	16	C_1	0.60	2.43	2.58

Table S4. The NPA charges (e) on the parent Si atoms, double-Gd atoms and total charges for the most stable isomers.

Clusters	NPA charges			
	Gd-1	Gd-2	Si_n	Total
3A	0.37	0.37	-1.74	-1
4A	0.48	0.43	-1.91	-1
5A	0.33	0.33	-1.66	-1
6A	0.30	0.30	-1.59	-1
7A	0.52	0.34	-1.86	-1
8A	0.36	0.41	-1.77	-1
9A	0.43	0.43	-1.86	-1
10A	0.47	0.47	-1.93	-1
11A	0.42	0.42	-1.83	-1
12A	0.45	0.45	-1.89	-1

Table S5. The valence electron configuration of each one of the two Gd atoms, magnetic moments (μ_B) of the Gd-4f orbital, total magnetic moments of the two Gd atoms, and total magnetic moments for the most stable isomers.

Clusters		Valence electron configuration	Magnetic moments of Gd atom (μ_B)		Total
			4f	Gd_2	
3A	Gd-1	$6s^{0.97}4f^{7.01}5d^{1.50}6p^{0.18}7s^{0.02}6d^{0.01}$	6.97	15.74	15
	Gd -2	$6s^{0.97}4f^{7.01}5d^{1.50}6p^{0.18}7s^{0.02}6d^{0.01}$	6.97		
4A	Gd -1	$6s^{1.20}4f^{7.01}5d^{1.08}6p^{0.22}7s^{0.04}6d^{0.01}8s^{0.01}$	6.99	14.73	15
	Gd -2	$6s^{1.08}4f^{7.01}5d^{1.24}6p^{0.23}7s^{0.04}6d^{0.02}7p^{0.01}8s^{0.01}$	6.99		
5A	Gd -1	$6s^{1.06}4f^{7.01}5d^{1.37}6p^{0.23}7s^{0.02}6d^{0.02}8s^{0.01}$	6.99	16.94	17
	Gd -2	$6s^{1.06}4f^{7.01}5d^{1.37}6p^{0.23}7s^{0.02}6d^{0.02}8s^{0.01}$	6.99		
6A	Gd -1	$6s^{0.92}4f^{7.01}5d^{1.50}6p^{0.29}7s^{0.03}6d^{0.02}8s^{0.01}$	6.98	15.66	15
	Gd -2	$6s^{0.92}4f^{7.01}5d^{1.50}6p^{0.29}7s^{0.03}6d^{0.02}8s^{0.01}$	6.98		
7A	Gd -1	$6s^{1.04}4f^{7.01}5d^{1.21}6p^{0.24}7s^{0.02}6d^{0.02}8s^{0.01}$	6.99	15.76	15
	Gd -2	$6s^{0.94}4f^{7.01}5d^{1.41}6p^{0.29}7s^{0.04}6d^{0.02}8s^{0.01}$	6.98		
8A	Gd -1	$6s^{0.92}4f^{7.01}5d^{1.42}6p^{0.30}7s^{0.04}6d^{0.02}7p^{0.01}$	6.98	15.73	15
	Gd -2	$6s^{0.98}4f^{7.01}5d^{1.32}6p^{0.30}7s^{0.02}6d^{0.02}$	6.99		
9A	Gd -1	$6s^{0.98}4f^{7.01}5d^{1.33}6p^{0.27}7s^{0.02}6d^{0.02}7p^{0.01}$	6.99	16.52	17
	Gd -2	$6s^{0.98}4f^{7.01}5d^{1.33}6p^{0.27}7s^{0.02}6d^{0.02}7p^{0.01}$	6.99		
10A	Gd -1	$6s^{0.93}4f^{7.01}5d^{1.33}6p^{0.28}7s^{0.03}6d^{0.02}7p^{0.01}$	6.99	15.62	15
	Gd -2	$6s^{0.93}4f^{7.01}5d^{1.33}6p^{0.28}7s^{0.03}6d^{0.02}7p^{0.01}$	6.99		
11A	Gd -1	$6s^{0.88}4f^{7.01}5d^{1.47}6p^{0.26}7s^{0.02}6d^{0.02}$	6.98	15.62	15
	Gd -2	$6s^{0.88}4f^{7.01}5d^{1.47}6p^{0.26}7s^{0.02}6d^{0.02}$	6.98		
12A	Gd -1	$6s^{0.43}4f^{7.02}5d^{1.84}6p^{0.29}6d^{0.02}$	6.98	14.96	15
	Gd -2	$6s^{0.43}4f^{7.02}5d^{1.84}6p^{0.29}6d^{0.02}$	6.98		

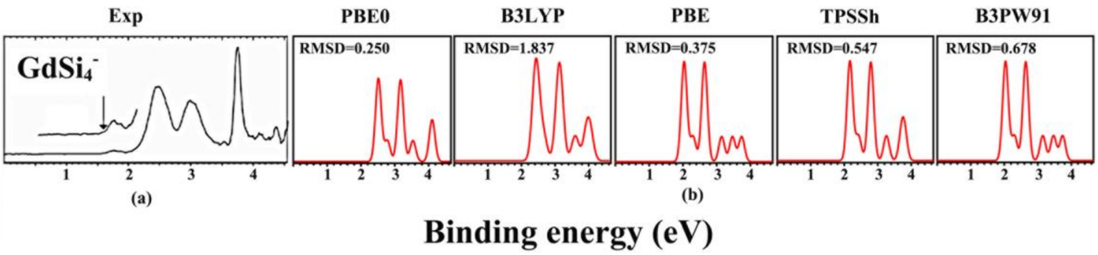

Figure S1. (a) Photoelectron spectrum of $GdSi_4^-$ measured at 266 nm (4.66 eV), the spectrum is taken from Ref. 20. (b) Simulated photoelectron spectra (PES) from the lowest-energy structures for $GdSi_4^-$ clusters at the PBE0/Gd/ECP28MWB//Si/6-311+G(d) level, together with B3LYP, PBE, TPSSh and BPW91 functionals for comparison. Each VDE was fitted with a full width at half-maximum (FWHM) of 0.20 eV to yield the simulated PES spectra.

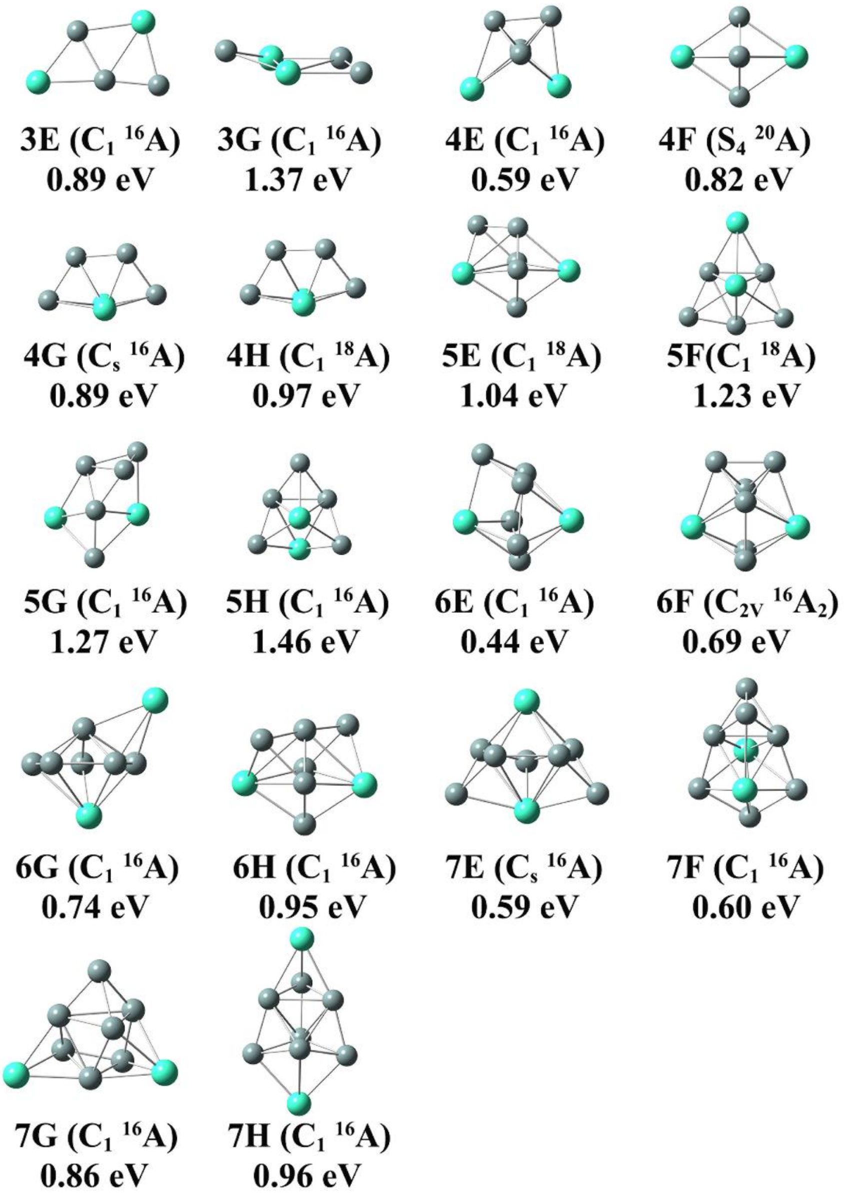

Figure S2. The various geometrical structures of $Gd_2Si_n^-$ (n =3-7) with relative energy, symmetry and electron state at the PBE0/Gd/ECP28MWB//Si/6-311+G(d) level. The gray and light green balls represent silicon and gadolinium atoms, respectively.

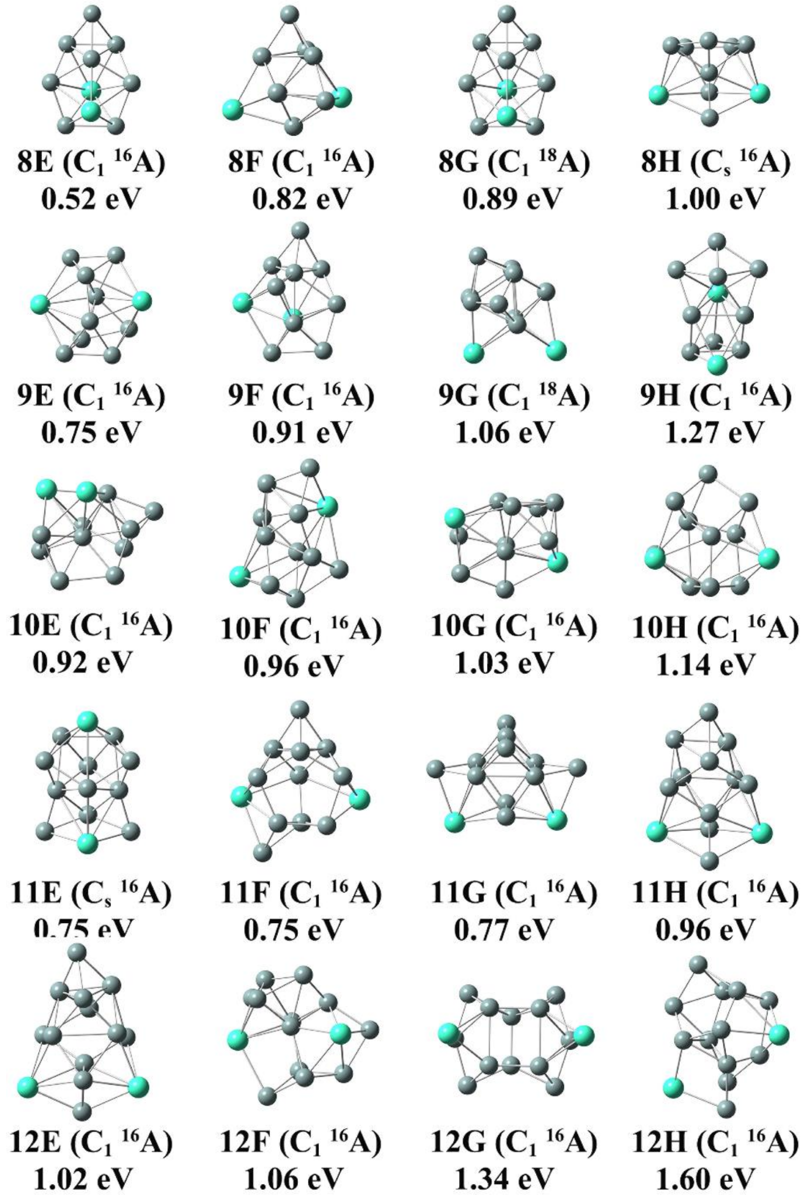

Figure S3. The various geometrical structures of $Gd_2Si_n^-$ (n =8-12) with relative energy, symmetry and electron state at the PBE0/Gd/ECP28MWB//Si/6-311+G(d) level. The gray and light green balls represent silicon and gadolinium atoms, respectively.

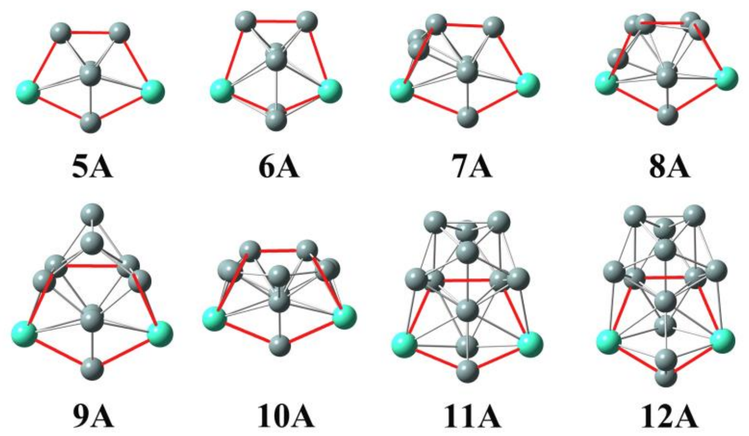

Figure S4. The growth behavior of $Gd_2Si_n^-$ (n =5-12) clusters.

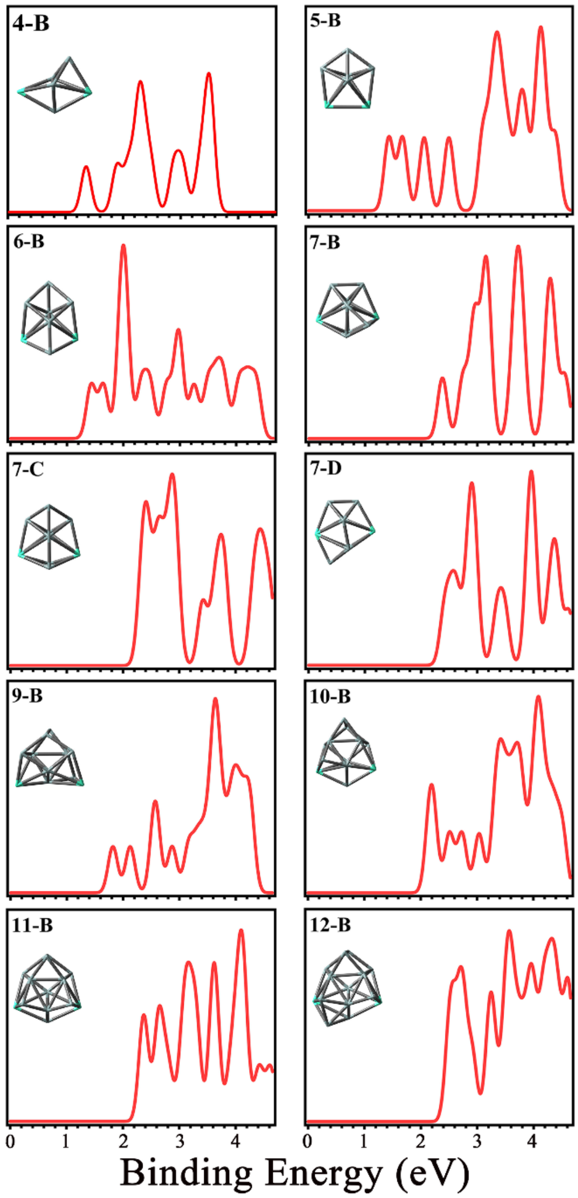

Figure S5. The simulated photoelectron spectra (PESs) for the assigned major clusters within 0.3eV at the PBE0/Gd/ECP28MWB//Si/6-311+G(d) level. The simulated PESs exhibit a full width at half maximum (FWHM) of 0.20 eV.

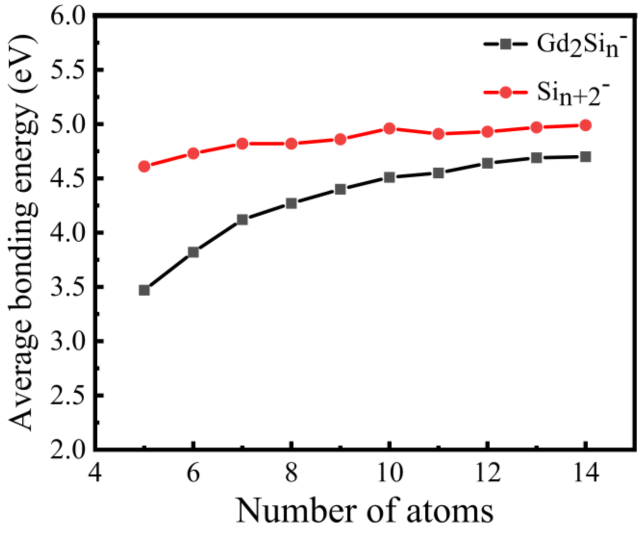

Figure S6. The average binding energies of $Gd_2Si_n^-$ (n =3-12) and Si_{n+2}^- at PBE0/Gd /ECP28MWB//Si/6-311+G(d) level.

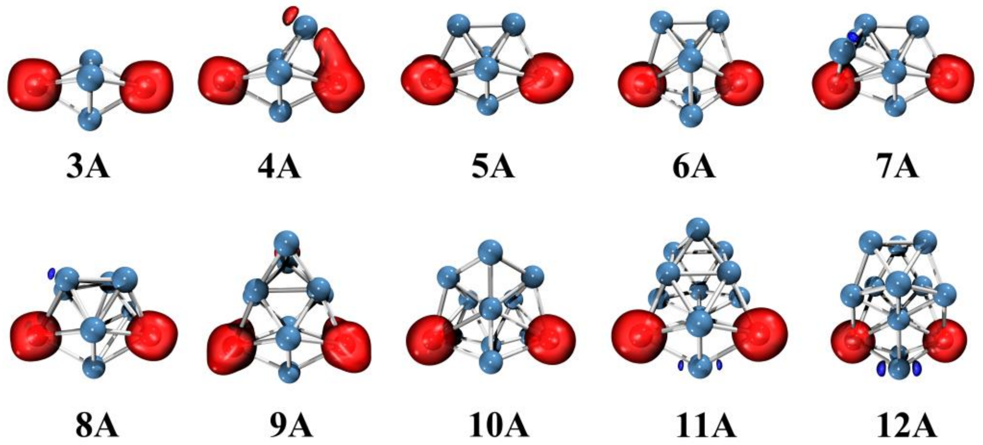

Figure S7. The spin-density (ρ_{alpha}–ρ_{beta}) isosurfaces of lowest-lying isomers of $Gd_2Si_n^-$ (n=3-12). The isosurface is set to ±0.02. The red and blue isosurfaces show that the spin density has positive and negative values, respectively.
